# Microenvironmental control of the ductular reaction: balancing repair and disease progression

**DOI:** 10.1038/s41419-025-07590-4

**Published:** 2025-04-04

**Authors:** Giovanni Sorrentino

**Affiliations:** 1 Department of Medical, Surgical and Health Sciences, University of Trieste, Trieste, Italy; 2 International Centre for Genetic Engineering and Biotechnology (ICGEB), Trieste, Italy

**Keywords:** Cell biology, Stem cells, Pathogenesis

## Abstract

The ductular reaction (DR) is a dynamic adaptive cellular response within the liver, triggered by various hepatic insults and characterized by an expansion of dysmorphic biliary epithelial cells and liver progenitors. This complex response presents a dual role, playing a pivotal function in liver regeneration but, paradoxically, contributing to the progression of liver diseases, depending upon specific contextual factors and signaling pathways involved. This comprehensive review aims to offer a holistic perspective on the DR, focusing into its intricate cellular and molecular mechanisms, highlighting its pathological significance, and exploring its potential therapeutic implications. An up-to-date understanding of the DR in the context of different liver injuries is provided, analyzing its contributions to liver regeneration, inflammation, fibrosis, and ultimately carcinogenesis. Moreover, the review highlights the role of multiple microenvironmental factors, including the influence of extracellular matrix, tissue mechanics and the interplay with the intricate hepatic cell ecosystem in shaping the DR’s regulation. Finally, in vitro and in vivo experimental models of the DR will be discussed, providing insights into how researchers can study and manipulate this critical cellular response. By comprehensively addressing the multifaceted nature of the DR, this review contributes to a more profound understanding of its pathophysiological role in liver diseases, thus offering potential therapeutic avenues for hepatic disorders and improving patient outcomes.

## Facts

- In response to liver injury, hepatic progenitor cells become activated through a process known as the ductular reaction (DR).
- DR plays a dual role in liver pathophysiology, supporting regeneration while also contributing to disease progression.
- DR is regulated by various signaling pathways and microenvironmental factors, along with the composition and mechanical properties of the extracellular matrix.
- DR is a promising therapeutic target for preventing liver disease progression.

## Open questions

- What specific factors determine whether DR plays a regenerative or pathological role in liver disease?
- What are the precise contributions of different signaling pathways on DR, and can selective modulation of these pathways provide therapeutic benefit?
- How do DR cells interact with different immune cell types to contribute to chronic inflammation and fibrosis in liver diseases?
- Can we identify reliable biomarkers from DR activity for non-invasive diagnosis and monitoring of liver disease progression?

## Introduction

**Fig. 1:**
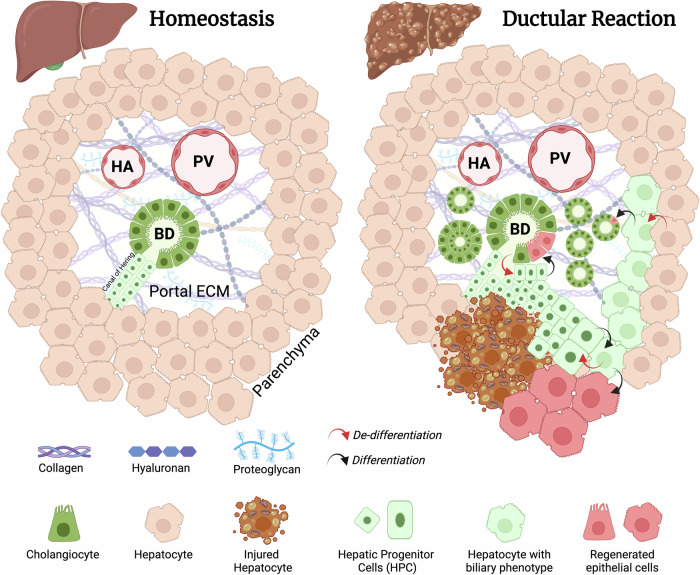
An overview of the DR in the liver. In response to liver injury, HPC in the canal of Hering or quiescent cholangiocytes in the portal space undergo activation, leading to the formation of reactive ductules that proliferate and eventually infiltrate the parenchyma. HPC can also derive from the liver parenchyma as a consequence of hepatocyte metaplasia characterized by acquisition of a biliary phenotype, while mature cholangiocyte can derive from peri-portal hepatocyte trans-differentiation. DR structures can take various forms, including multiple small ducts, multilayered ductular structures, or HPC cords infiltrating the hepatic parenchyma. HPC can differentiate back to cholangiocytes and hepatocytes in order to regenerate the tissue. HA hepatic artery, PV portal vein, BD bile duct.

The term Ductular reaction (DR) was coined by Hans Popper in 1957 and refers to a complex cellular response characterized by bile duct hyperplasia observed in the liver during various hepatic insults and pathological conditions [1]. The DR is a dynamic process that occurs in response to bile ducts injury or parenchymal damage, mainly when regenerative capacity of hepatocytes is impaired or overwhelmed, such as in chronic liver diseases. It serves as an adaptive mechanism aimed at restoring tissue integrity and functions through a process of progenitor-dependent epithelial regeneration [2]. This process mainly involves hepatic progenitor cells (HPC) mobilization from their quiescent state or activation of biliary epithelial cells (BEC, aka cholangiocytes) undergoing de-differentiation toward HPC and proliferation (Fig. 1). HPC were first described in rat liver in 1937 by Kinosita as cholangiocyte-like cells belonging to what was termed by Farber later in 1956 the ‘oval cell compartment’, due to the morphological feature of clusters of small ovoid cells with high nuclear:cytoplasmic ratio [3]. HPC reside in the portal areas of the liver, in close proximity to the bile ducts and canals of Hering (Fig. 1). HPC are a heterogeneous and highly plastic population of cells with stem/progenitor properties displaying the ability to proliferate, self-renew and terminally differentiate into both BEC and hepatocytes, thus acting as facultative bi-potent epithelial stem cells [4]. Furthermore, in response to injury, certain BEC and HPC may undergo senescence caused by chronic inflammation, where these metabolically active cells no longer respond to mitogenic stimuli but instead activate a pro-inflammatory response known as “senescence-associated secretory phenotype” (SASP), releasing soluble factors similar to those produced by inflammatory cells [5]. The origin of DR cells is still debated and likely depends on the nature of the liver injury. Indeed, in different pathologic settings, they have been shown to originate either from HPC; de-differentiation of adult BEC; or from hepatocyte ductular metaplasia [6] (Fig. 1). Histologically, DR is characterized by the activation, proliferation, and eventually differentiation of HPC, leading to the formation of ductular-like structures within the portal area or the liver parenchyma [7]. The DR can present in various forms, including the emergence of multiple small ducts within the portal area with variable luminal definition; the formation of multilayered ductular structures; or the presence of “cords” of BEC lacking a lumen and infiltrating the hepatic parenchyma facing the portal mesenchyme (Fig. 1). These proliferating ductules exhibit characteristics of both cholangiocytes and hepatocytes, but displaying mainly markers of BEC, such as cytokeratins (e.g., CK7, CK19) and epithelial cell adhesion molecule (EpCAM).

**Fig. 2:**
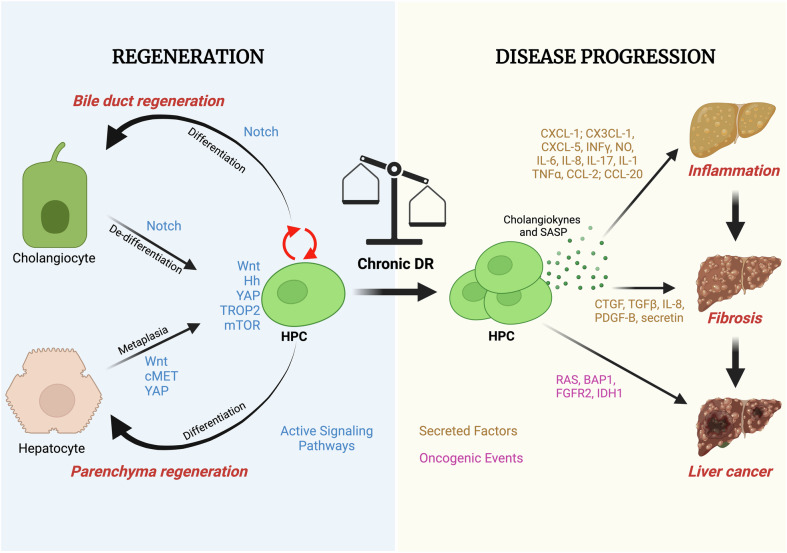
Duality of DR response: regeneration and disease progression. An overview of the opposing functions exhibited by DR cells in regeneration (left) or disease progression (right). The figure underscores the complexity of these cellular responses and emphasizes key molecular pathways governing these divergent functions. On the left side of the figure, DR cells act as agents of tissue repair and regeneration. They engage in various processes that collectively contribute to the restoration and renewal of damaged tissues. Conversely, the right side of the figure illustrates the disease-related functions of chronically-activated DR cells. In this context, DR cells are implicated in processes that exacerbate diseases. The molecular pathways associated with this function involve release of cholangiokynes from proliferating HPC cells or SASP from senescent HPC that can enhance inflammation, facilitate fibrosis, and promote liver disease progression, including cancer.

The DR has both beneficial and detrimental effects on liver pathology, depending on the type of injury and environmental factors. Although the effective contribution of DR in liver regeneration remains largely unproved in humans, it is believed to serve as an attempt to restore liver function and promote tissue repair (Fig. 2). On the contrary, in certain chronic liver diseases such as cholangiopathies, alcoholic hepatitis, Metabolic dysfunction-Associated SteatoHepatitis (MASH), and cirrhosis, persistent and dysregulated DR has been demonstrated to contribute to inflammation, fibrogenesis, and liver dysfunction [7] (Fig. 2). For this reason, DR has recently emerged as a promising pharmacological target for chronic liver diseases, offering opportunities for therapeutic interventions aimed at preventing the progression of these conditions [8].

### Ductular reaction in liver regeneration

In normal conditions, hepatic injury leads to the death of some hepatocytes, allowing healthy hepatocytes to proliferate and replace the lost cells. In this context, DR contribution to parenchymal regeneration is minimal. However, in cases of chronic injury, when hepatocyte proliferation is compromised due to mechanisms such as senescence, epigenetic/signaling abnormalities, or other mechanisms, BEC or HPC plastically re-enter the cell cycle, expand, and ultimately acquire biliary or hepatocyte identity, thus supporting parenchymal repair and re-establishment of liver functions (Fig. 2). In support to this hypothesis, careful lineage-tracing studies have shown that, HPC or BEC terminally differentiate into functional hepatocytes in mice with liver injury [9–11]. Loss of β1-integrin in hepatocytes of mice with liver damage triggers a DR, resulting in the significant emergence of BEC-derived hepatocytes that coordinate a substantial parenchymal regeneration [12]. Loss of β-catenin in hepatocytes of mice placed on the CDE diet develops severe liver injury that stimulates BEC to differentiate into hepatocytes and drive regeneration [13]. Of note, DR-derived hepatocytes are characterized by higher proliferative capability, less apoptosis, a lower proportion of highly polyploid nuclei and ultimately are able to eliminate DNA damage, making DR an interesting target for regenerative medicine [9–15]. In addition, HPC transplanted in mouse and human livers demonstrated regenerative capacity in vivo, thus highlighting their therapeutic potential for cell therapies [14, 16]. Moreover, BEC-derived hepatocytes upon chronic injury gain a Hnf4α^+^CK19^+^ bi-phenotypic state in periportal regions and fibrotic septa, which can also be detected in cirrhotic human livers [9].

DR can be reversed when the causative trigger is eliminated through mechanisms involving apoptosis of ductular structures leading to improvement of the disease progression. On the contrary, in the presence of chronic liver diseases, the DR transitions into a chronic state, significantly limiting its regenerative capacity for the liver parenchyma and activating a chronic wound-healing response characterized by pro-inflammatory and fibrogenic signals that promote disease progression (Fig. 2). Thus, the impact of DR on liver diseases is a double-edged sword and understanding the intricate cellular and molecular mechanisms underlying the DR during liver regeneration is essential for developing effective strategies to enhance liver regeneration in various liver diseases and conditions.

### Ductular reaction in chronic liver diseases

Although DR can support the activation and differentiation of HPC and aid in the repair of liver injury in some experimental setting, its effect in chronic liver disease may not consistently yield positive outcomes, and, on the contrary, contribute to the occurrence and progression of inflammation and liver fibrosis (Fig. 2). In line with this notion, DR occurs in association with a large spectrum of human liver diseases and in numerous experimental animal models of hepatic injury and, importantly, selective ablation of DR cells prevents disease progression in mouse models of cholestasis [17, 18]. Chronic viral hepatitis, particularly hepatitis B (HBV) and hepatitis C (HCV) infections, are major causes of DR, which plays a significant role in the progression of these diseases [19, 20]. Here, the DR is activated by viral proteins from HBV and HCV and observed in areas of liver tissue where hepatocyte necrosis and inflammation occur [21]. For example, HBV X protein (HBx) and HCV core protein can activate signaling pathways involved in HPC activation and proliferation [22].

Alcohol-Associated Liver Disease (ALD) is a consequence of chronic and excessive alcohol consumption which leads to hepatocyte damage, inflammation, and oxidative stress, resulting in chronic liver injury. In response to alcohol-induced liver injury, the DR is initiated as a reparative process but progresses as a maladaptive wound-healing response which correlates with disease severity, prognosis and mortality [23–25].

Metabolic dysfunction-Associated Steatotic Liver Disease (MASLD) encompasses a spectrum of conditions characterized by the accumulation of fat in the liver in the absence of excessive alcohol consumption. MASH represents a more advanced stage of MASLD characterized by liver inflammation and ballooning degeneration of hepatocytes. The DR is frequently observed in MASLD and is implicated in the progression of MASLD to MASH. In this pathological context, the DR is particularly evident in parenchymal areas of liver inflammation and fibrosis and may derive from hepatocyte ductular metaplasia. Of note, correlative studies have demonstrated that DR associates with fibrosis stage in MASH patients [26–29].

Chronic cholestatic liver diseases such as Primary biliary cholangitis (PBC), Primary sclerosing cholangitis (PSC) and Biliary atresia (BA) are characterized by inflammation and damage of the bile ducts. In such cholangiopathies, the DR is an important component of the pathophysiology and has a prognostic role by predicting response to therapy and disease progression [30–33].

The DR has been recognized as a precursor lesion for hepatocellular carcinoma (HCC), cholangiocarcinoma (CCA) and mixed HCC-CCA, the most common forms of primary liver cancer [34, 35]. Lineage-tracing studies of EpCAM-expressing biliary proliferating cells, demonstrated that these cells can develop into HCC [36]. Furthermore, lineage-tracing experiments using Mdr2-KO mice revealed that HPC serve as the source of mixed HCC-CCA. In this model, chronic liver inflammation and fibrosis triggered HPC activation, and the ablation of HPC significantly reduced mixed HCC-CCA formation [37].

The involvement of HPC in intrahepatic CCA has also been shown and mechanistically linked to specific genetic mutations. Mutant IDH (Isocitrate dehydrogenase 1) proteins disrupt hepatocyte differentiation by suppressing HNF-4α, a master regulator of hepatocyte identity, thereby driving HPC proliferation. The combined action of IDH and Kras mutations promotes the expansion of HPC, the development of premalignant biliary lesions, and progression to metastatic CCA [38].

The clinical relevance of DR cells as a cell of origin for CCA is further supported by studies in PSC where the chronic activation of DR cells provides an ideal setting for the acquisition of genetic alterations or epigenetic changes and the development of neoplastic features. The progressive accumulation of genetic and epigenetic changes in DR cells can lead to the formation of dysplastic nodules, which can further progress to liver cancer. Consequently, PSC patients exhibit a significantly elevated lifetime risk of developing CCA, estimated to exceed 10%. This clinical evidence strongly links PSC, DR, and CCA, highlighting the oncogenic potential of DR cells and the critical interplay between chronic liver injury and biliary tumorigenesis [39]. Experimental models have further demonstrated the tumorigenic potential of DR cells in vitro. Organoids derived from HPC, which recapitulate key features of the DR, have been genetically modified to harbor cancer-associated mutations such as BAP1 loss and FGFR2 fusions. These engineered organoids exhibit abnormal proliferation and differentiation defects, mimicking early tumorigenic events. When transplanted orthotopically into mouse models, these organoids reliably progress to CCA, directly demonstrating the oncogenic potential of genetic alterations in DR cells [40, 41].

In conclusion, while DR plays a crucial role in liver regeneration, its persistent activation in chronic liver diseases contributes to fibrosis, inflammation, and carcinogenesis, making it both a potential therapeutic target and a key factor in disease progression (Fig. 2).

### Origin and fate of ductular reaction cells in different etiologies

DR cellular origin and fate can diverge based on the underlying injurious stimuli and disease context. In cholestatic conditions, such as PBC, PSC, BA and bile duct obstruction, persistent damage to cholangiocytes triggers extensive ductular proliferation, which aims to restore the surface area of bile drainage. The origin of DR cells in these conditions primarily includes HPC located in the canals of Hering and cholangiocytes that re-enter the cell cycle. Histologically, these cells express biliary markers such as CK19 and CK7. The fate of DR cells in cholestatic injuries is predominantly biliary differentiation, maintaining a ductular phenotype to repair damaged biliary ducts, with no significant differentiation into hepatocytes [2] (Fig. 2).

In hepatocellular injuries, including chronic HBV, HCV, alcohol-related liver disease, or MASH, DR is less intense and DR cells originate from HPC in the periportal regions and aim to re-establish the connection between damaged hepatocytes, the destroyed canaliculi, and the biliary tree. These cells exhibit the capacity to differentiate into both cholangiocytes and hepatocytes, depending on the extent of injury and local microenvironmental cues. In experimental models of widespread hepatocyte necrosis or dysfunction, HPC-derived cells can shift toward a hepatocytic fate, replenishing damaged parenchyma. However, ongoing inflammation and fibrosis often restrict their hepatocytic potential [2] (Fig. 2).

In toxin-induced liver injuries, such as those caused by carbon tetrachloride (CCl_4_) or acetaminophen (APAP) in experimental models, DR cells originate from both HPC and proliferating cholangiocytes. The fate of DR cells in these models varies with the severity of the injury. While biliary differentiation is predominant, there can also be hepatocytic differentiation under conditions of severe parenchymal damage.

In ischemia/reperfusion injuries, DR cells originate mainly from HPC in the periportal regions, which become activated in response to localized necrosis. These cells generally remain confined to a biliary phenotype, as the injury is often restricted to periportal areas where biliary structures dominate. Of note, in ischemia and fatty liver disease, DR can be seen also in pericentral location, caused by hepatocyte trans-differentiation [2] (Fig. 2).

Across all these conditions, the local microenvironment plays a crucial role in shaping both the origin and fate of DR cells. These differences are evident at the molecular level, as demonstrated by a recent study analyzing the transcriptomic profiles of HPC derived from livers affected by hepatitis C virus infection and PSC, which serve as models for hepatocellular and biliary injury, respectively. This study revealed notable disparities in the expression of more than 300 genes among the DR cells in these two diseases, providing insights into how these variations affect the recruitment and localization of inflammatory cells [42].

Understanding the disease-specific properties of DR is not a trivial distinction but a critical step toward precision medicine in hepatology. However, the molecular, cellular, and inflammatory differences in DR across various liver diseases remain poorly defined, and their impact on disease progression is not fully understood. Further research in this area could help unlock new treatment paradigms, making it a high-priority open question in liver pathology.

### Cellular and molecular mechanisms of ductular reaction

The DR involves intricate cellular and molecular mechanisms regulated by a network of signaling pathways and microenvironmental cues that drive the activation, proliferation, and differentiation of HPC. Notch signaling plays a crucial role in the fate determination of HPC, promoting their differentiation into cholangiocyte-like cells. DR in human BA and cholestasis induced by 3,5-diethoxycarbonyl-1,4-dihydrocollidine (DDC) in mice are associated with Notch signaling activation, which induces the expression of biliary markers and drives the formation of ductular structures. Moreover, inhibition of recombination signal binding protein immunoglobulin kappa J (RBP-jκ), a common DNA-binding partner of Notch receptors, reduces DR and biliary fibrosis in DDC mice [43]. Similarly, it has been found that the Notch-RBPJ signaling axis regulates biliary regeneration by coordinating the fate decision of HPC toward cholangiocytes [44] while Notch inhibition in steatohepatitis models reduced DR and cholestatic liver fibrosis [45, 46]. During biliary regeneration, expression of Jagged 1 (a Notch ligand) by myofibroblasts promoted Notch signaling in HPC and thus their biliary specification to cholangiocytes [47]. Finally, in cholangiocytes, CCN1 activated NF-κB through integrin α_v_β_5_/α_v_β_3_, leading to *Jag1* expression, JAG1/NOTCH signaling, and proliferation [48].

The Wnt/β-catenin signaling is also implicated in promoting HPC proliferation and expansion [49]. Upon partial hepatectomy, in the absence of β-catenin a dramatic reduction in HPC number has been observed, thus demonstrating a crucial role of Wnt/β-catenin in HPC biology [50]. Wnts regulate HPC proliferation and hepatocyte-to-cholangiocyte metaplasia. Specifically, Wnt7b is induced in DR cells in DDC mice [51]. However, macrophage-derived Wnt proteins have been shown to prevent DR by opposing the Notch signaling [47, 52]. The Wnt target gene Leucine-rich repeat-containing G-protein coupled receptor 5 (Lgr5) is activated in DR cells that appear near bile ducts upon damage, characterized by robust activation of Wnt signaling. Of note, Lgr5^+^ cells are able to regenerate both hepatocytes and cholangiocytes in vivo [53]. However, dysregulation of Wnt/β-catenin signaling can lead to aberrant HPC expansion and contribute to liver pathologies [54], including fibrosis and HCC.

The Hedgehog (Hh) signaling is well-known to control proliferation of fetal liver progenitors and adult HPC activation, proliferation, and differentiation [55]. In MASH, ductular cells express OPN, downstream of the Hh pathway, which contributes to hepatic stellate cells (HSC) activation and fibrogenesis [56, 57]. In PBC, the Hh pathway is activated in HPC during the fibroproliferative response to chronic cholestatic liver injury [58]. Furthermore, markers of canonical Hh signaling in human cirrhosis were predominantly found to be confined to the DR cells and the Hh transducer Gli supports HPC survival [59] and regeneration during chronic injury [60].

Recent studies have shown that the Hippo pathway plays a pivotal role in the activation of HPC and is instrumental to activate a DR response. Upon liver injury, the Hippo nuclear transducers YAP/TAZ translocate into the nucleus in HPC [61–65] and initiate a transcriptional program associated with acquisition of progenitor features, through activating the Notch signaling [66–68]. Conversely, inhibition of YAP/TAZ can suppress cholangiocyte differentiation and bile duct morphogenesis and YAP/TAZ deletion in adult bile ducts causes severe defects and delay in liver regeneration.

TROP2, is expressed exclusively in HPC, establishing TROP2 as a reliable marker to distinguish DR cells from normal cholangiocytes [69]. Moreover, single-cell RNA sequencing (scRNAseq) from normal liver tissue identified a TROP2^int^ progenitor population with strong potential to form bipotent liver organoids in vitro [70]. Recombinant hepatic growth factor (HGF) is known to promote regeneration in rodents after partial hepatectomy [71]. The HGF/c-MET axis promotes the migratory and invasive phenotype of HPC as well as DR and progenitor-mediated liver regeneration [72, 73]. Of note, c-Met stimulation promotes the trans-differentiation of hepatocytes toward biliary cells [74].

Cell metabolism plays a crucial role in DR cells, as an effect of the increased demand for energy and biosynthetic precursors to support the proliferation of HPC. Additionally, changes in cellular metabolism can influence the fate and plasticity of HPC, determining whether they differentiate into hepatocytes or cholangiocytes. In this context, a recent CRISPR screen identified the metabolic master regulator mammalian target of rapamycin (mTOR) as a crucial player in HPC growth in vitro [62]. Furthermore, lipid overload has been found to prime the reprogramming of a subset of BEC to a “progenitor” state in early stages of MASLD [75].

In addition to these well-known signaling pathways, other key proteins, including CD44 [76]; CD133(PROM1) [18]; SOX9 [77]; and OV6 [78], seem to have a prominent role in DR and are considered markers of HPC.

Overall, the cellular and molecular mechanisms of DR activation are complex and tightly regulated processes and the interplay between different signaling pathways governs the activation, proliferation, and differentiation of HPCs, ultimately shaping the DR and its implications in liver pathology (Fig. 2).

### Inflammatory and fibrogenic functions of ductular reaction cells

Cholangiokines represent a group of secreted factors released by quiescent, senescent and proliferating cholangiocytes or HPC during DR and include cytokines, chemokines and growth factors (Fig. 2) [79]. When activated, HPC display a distinct secretory profile that significantly influences the surrounding microenvironment by regulating the recruitment of immune cells and the migration and activation of mesenchymal cells [80]. Cholangiokines secretion is influenced by different factors such as tissue inflammation, infection, and metabolic dysregulation and actively maintain the portal area’s microenvironment contributing to immune response and inflammation [81]. Cholangiocytes’ secretory activity functions in autocrine, paracrine, and endocrine manners to uphold biliary homeostasis and influence other cell types, including hepatocytes, HSCs, and macrophages [82], ultimately supporting different pathological states [83]. IL-6 and IL-17, secreted by reactive cholangiocytes, facilitate differentiation of T-helper (Th) cells. Histological and single cell RNA sequencing data from individuals with PSC indicate the accumulation of Th cells in the liver, specifically proximal to DR cells [84–86]. DR cells, in turn, release CX3CL1 and CXCL-1, which attract monocytes and T cells [87, 88]. Upon biliary injury, reactive cholangiocytes secrete TNFα and IL-6 [89]. The former activates naïve and effector T cells, while the latter regulates B cell terminal differentiation and the balance between Th17 and Treg cells. The anti-inflammatory drug dexamethasone efficiently prevents HPC activation via TNFα and IL-6 inhibition [90]. Human cholangiocytes also secrete CCL-20 and CCL-2, promoting infiltration of Th17 cells, monocytes, and dendritic cells into portal tracts [91, 92]. Moreover, substantial biliary damage triggers pronounced CX3CL1 release, attracting natural killer (NK) cells [93]. Immune factors released by DR cells, including IL-1b, IL-6, IL-23, CXCL-1/2/3/6/8, CCL-2/20, are overexpressed in PSC patients [85]. The inflammatory mediator CXCL-5 is released by DR cells and is overexpressed in patients with alcoholic hepatitis [94]. IFNγ prompts a shift in cholangiocyte cytokine secretion, transitioning from acute to chronic inflammation and decreases IL-8 secretion while elevating the release of diverse cytokines such as MCP-1, monokine, ITAC, and IP10. Additionally, both IFNγ and IL-6 stimulate nitric oxide (NO) production in cholangiocytes by upregulating nitric oxide synthase-2 (NOS-2) expression [95–98]. IFNα reduces the number of HPC upon CDE diet [99]. During Helicobacter bilis or flukes infection, cholangiocytes proliferate and substantially release IL-6 and IL-8, initiating the activation of innate mucosal immunity against microorganisms [100, 101].

Beyond the role in portal inflammation, DR cell’s secretome orchestrates dynamic interaction between reactive ductular cells and portal myofibroblast cells, known as epithelial–mesenchymal crosstalk, that directly stimulates hepatic stellate cells (HSC) activation, collagen deposition and fibrosis. Of note, previous analyses of chronically diseased human livers have demonstrated a correlation between the rate of DR and the severity of fibrosis [26, 102–105]. DR cells play a crucial role as a source of the fibrogenic connective tissue growth factor (CTGF), which is also triggered by YAP in response to tissue stiffening during fibrosis progression [106, 107]. Recent studies have unveiled a prominent role of cholangiocytes in biliary fibrosis, particularly through their communication with HSC, driven by TGFβ-mediated signaling and the epigenetic activation of a fibrogenic gene network involving H3K9 acetylation [108]. Moreover, recent data indicate that portal fibroblasts trans-differentiation into myofibroblasts, driven by the MCP-1/CCL2 axis, acts in a paracrine loop with BEC [109]. TGF-β2 expression in DR cells is evident in fibrotic liver and correlate with fibrosis activity and bile duct proliferation [110]. IL-8 levels are high in the bile of PSC patients, and stimulate cholangiocyte proliferation and expression of pro-fibrotic genes [111]. During chronic cholestasis, BEC have the capacity to produce platelet-derived growth factor-B (PDGF-B) which activates HSC leading to apoptosis susceptibility and fibrogenesis [112]. These data also demonstrated the efficacy of the BH3 mimetic A-1331852 in reducing both senescent BEC and activated stromal fibroblasts, thereby ameliorating biliary fibrosis in an experimental PSC model [113]. In a recent study Zhang et al. have described the pivotal role of NF-κB-inducing kinase (NIK) in orchestrating DR and fibrogenesis in chronic liver diseases [114]. The upregulation of biliary NIK, mediated by insults like DDC, ANIT, or BDL treatment, plays a crucial role in promoting DR, liver injury, inflammation, and fibrosis. NIK’s influence on immune cells and HSC via cholangiokines contributes to the proinflammatory and profibrotic milieu. In both BDL and Mdr2 knockout (Mdr2-/-) mouse models, exosomes enriched with lncRNA-H19 and secreted by cholangiocytes, were readily taken up by HSC, accelerating liver fibrosis. Additionally, these exosomes were found to enhance the trans-differentiation of primary mouse HSC, promoting their activation and inducing proliferation and extracellular matrix production [115]. In cholestatic liver injury, PROM1-expressing HPC promote biliary fibrosis associated with activation of myofibroblasts [18].

Integrin αvβ6, up-regulated in proliferating HPC during tissue injury, is essential for their function by activating TGFβ1 and promoting their adhesion to fibronectin [116, 117]. Inhibiting αvβ6 using the 3G9 antibody or genetic disruption not only hampers HPC activation in chronic biliary injury mouse models but also provides protection against liver fibrosis and tumorigenesis. The neuroendocrine secretin signaling promotes DR by stimulating cholangiocyte proliferation through PKA-ERK1/2 axis, thus contributing to increased cholangiocyte expansion observed in PSC patients [118]. Moreover, after liver injury, secretin produced by cholangiocytes reduces microRNA 125b and let7a levels, resulting in up-regulation of VEGF and NGF [119] and modulation of TGF-β1, which can be inhibited by secretin receptor antagonism, thus preventing fibrosis progression [120].

In conclusion, HPC orchestrates liver injury responses through diverse secretory phenotypes, influencing immune cell recruitment, inflammation, stromal activation, and fibrosis thus highlighting their crucial role in liver pathogenesis and offering potential therapeutic targets for preventing cholangiopathies and fibrosis.

### Role of portal ECM in ductular reaction

**Fig. 3:**
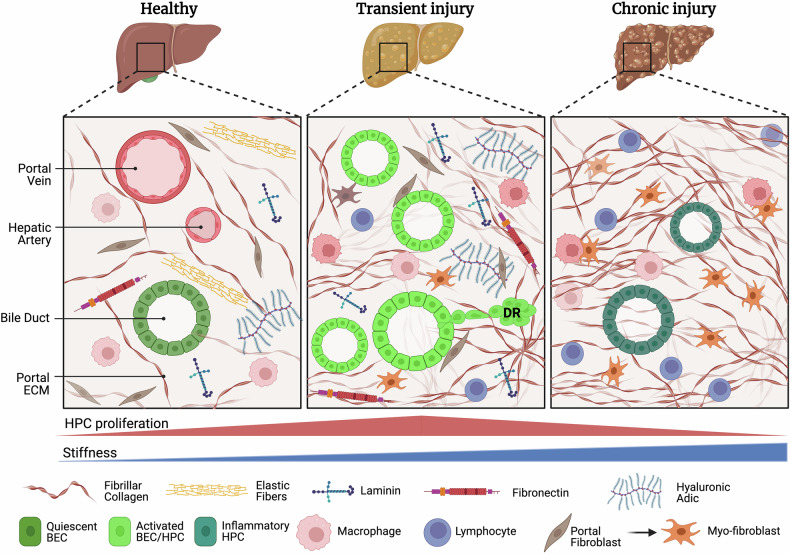
ECM and tissue mechanics in DR across disease progression. The dynamic interplay between the ECM and DR during disease progression. On the left (healthy), the tissue under normal physiological conditions. The portal ECM is depicted with a sparse distribution of collagen and elastic fibers, adhesive peptides and proteoglycans, which mirrors its typical composition in health where BEC are in a quiescent state. In the middle (transient injury): the ECM undergoes modifications, resulting in a mild accumulation of collagen fibers and a subtle increase in stiffness. These changes in ECM properties support a vigorous DR promoting activation and marked proliferation of HPC. In the right (chronic damage): during portal fibrosis collagen accumulation increases significantly, leading to an aberrantly high level of tissue stiffness which reduces the HPC replicative potential and promotes an inflammatory phenotype.

The specialized niche where the HPC get activated and from which DR expands is the portal zone, which consists of a bile duct, a branch of the hepatic artery, and a branch of the portal vein, commonly referred to as “portal triad” (Figs. 1 and 3). The portal triad is structurally supported by a complex network of ECM proteins that build up a specialized matrix called the “space of Mall” that resembles that of fetal and neonatal liver, containing collagen fibers, fibronectin, proteoglycans, laminin and playing instructive roles in determining HPC fate. It is generally accepted that HPC are located close to the canals of Hering within peri-portal zone 1, a link between the hepatocyte canalicular system and the biliary tree, and this location is consistent with their bi-potential property. Spatially, proliferative cholangiocytes are mainly located in the peripheral ductules and a classification of DR based on its geometrical configuration has been proposed Desmet [121, 122]. Type 1 (typical) DR, remains confined within the portal area and originates from pre-existing cholangiocytes; type 2 (atypical) A, mostly periportal, caused by HPC or ductal metaplasia of peri-portal hepatocytes; type 2B, occurs in the parenchyma hypoxic areas; and type 3 DR, caused by activation and proliferation of HPC located in the canals of Hering in response to massive hepatocyte necrosis, which extends further into the parenchyma [123]. Clerbaux further extended this classification based on grade of invasiveness into the parenchyma: type 1 non invading DR; type 2 invades the parenchyma within approximately 80 µm beyond the portal mesenchyme; type 3 invasive DR [123]. It has been suggested that invasive DR might play a role in restoring hepatobiliary junctions and ensuring bile excretion during hepatocellular injury [122, 123].

Fibrillar collagens are abundant in the portal region and provide tensile strength to the portal space and help maintain its structural integrity (Fig. 3). The portal space provides a favorable environment for BEC to acquire an activated state in response to tissue damage, in terms of both biochemical and physical signals. In healthy livers, Type I and III collagens constitute 95% of the collagens whereas type IV, V and VI collagens constitute 1%, 2–5% and 0.1%, respectively [124]. The portal ECM is composed mainly of collagen I and mature elastic bundles with a sharp edge facing the limiting plate, the first line of hepatocytes facing the portal region. The peri-sinusoidal ECM, instead, is made mainly of collagen III with no elastic fibers [125]. Compelling data from Leclerq’s lab have elegantly demonstrated the requirement of an ECM-rich microenvironment for efficient HPC expansion and lobular invasion [126] and, in particular, collagen I and laminin seem to be required to support an efficient DR [126, 127]. Therefore, HPC are embedded in a defined fibrillar ECM matrix that functions as a scaffold to support HPC activation to drive their infiltration into the parenchyma during DR (Fig. 3) [126].

Non-collagenous glycoproteins are also abundant in the portal ECM and include laminin, entactin, fibronectin, nidogen and tenascin [128]. These ECM proteins play a crucial role in both DR onset and propagation. Signalling via integrin α6β1 is suggested to play a critical role in HPC survival and growth, possibly through interaction with laminin and fibronectin in the portal ECM and activation of the ERK1/2 and p38 MAPKs pathways [129, 130]. Moreover, the activation and spreading of DR cells into the hepatic parenchyma is closely associated with the accumulation of laminin, fibronectin and collagen during hepatic regeneration [126, 131–133]. However, ECM proteins can also control the fate of HPC with laminin and collagen IV promoting their self-renew, while fibronectin and collagen I supporting their quiescence/differentiation state [128, 133]. Of note, different ECM compositions across liver zones might influence HPC behavior. For example, the distinct composition of the discontinuous basement membrane at the canal of Hering and parenchyma interface, characterized by laminin-rich biliary ECM and loosely aggregated type IV collagen fibers in adjacent hepatocytes’ space of Disse, may influence the unique niche functionality in this region within the hepato-biliary linkage context [134]. CD44, a transmembrane glycoprotein family, is expressed on HPC’s surface and binds to ECM components such as the glycosaminoglycan hyaluronic acid (HA), collagen type I, fibronectin, and laminin. CD44 aids HPC proliferation and infiltration [76, 135]. Moreover, CD44v6, a Met co-receptor, enhances HPC responses to HGF, suggesting a role in HPC-HGF interactions.

Finally, proteoglycans and their attached glycosaminoglycan chains have a role in the regulation of liver ECM properties through their water retention capabilities [136] and serve as a reservoir in portal zone for various growth factors and cytokines potentially impacting the DR response.

In summary, the periportal zone and its peculiar biochemical composition are vital for HPC activation and expansion during the DR.

### Regulation of ductular reaction by tissue mechanics

The liver is a highly complex organ with unique tissue mechanics, particularly in the portal space, that plays a critical role in regulating DR [137]. Tissue mechanics are constantly modulated by mechanical forces resulting from blood flow, ECM elasticity, and cell contractility. These biomechanical cues have been associated with the activation of specific signaling pathways (e.g., ERK, Hippo, Notch.) and are correlated with growth factor presentation [138–140]. Although healthy liver consists of relatively soft tissue with stiffness values lower than 1 kilopascal (kPa) in mice, local stiffness has been shown to be greater near the periportal region compared to the parenchymal and peri-central area [141], indicating that HPC are exposed to a peculiar mechanical niche able to potentially regulate their activation (Fig. 3). In addition, liver stiffness can substantially increase progressively during liver fibrosis, due to aberrant accumulation of collagen I fibers and such conditions are often associated with increased DR, suggesting that matrix remodeling can directly control HPC activation/mobilization [142, 143]. However, as the disease progresses, the liver stiffness can escalate to as high as 20 kPa, a pathological condition associated with reduced activation and proliferation of HPC. This evidence suggests that during chronic liver diseases, HPC are subjected to an increasingly rigid ECM that initially promotes their activation until a threshold elasticity is reached. Beyond this point, the abnormally stiff ECM inhibits HPC expansion, leading to detrimental effects on their regenerative capacity (Fig. 3). In line with this, Kallis et al. used the metalloprotease-resistant collagen Ia1(r/r) mouse, which has an exaggerated fibrotic response to liver injury, to investigate the link between ECM stiffness and HPC response and found that these mice displayed significantly reduced HPC response in chronic liver injuries compared to wild-type, associated with persistent collagen I and impaired laminin deposition [144]. Building on this ground, we have recently developed a standardized method to study the behavior of HPC in a defined mechanical environment, using mechanically-tunable synthetic polyethylene glycol (PEG) hydrogels. We were able to accurately model the aberrant mechanical properties of the fibrotic liver, providing evidence that, while physiological elasticity of the microenvironment is required for full HPC activation, an aberrantly stiff ECM negatively impacts liver progenitor proliferation [145]. Collectively, this evidence aligns with the notion that a physiologically elastic ECM is required for the complete induction of a DR. Studies on the molecular mechanisms by which ECM mechanics impact HPC biology suggest a key role of the nuclear mechano-transducers YAP/TAZ. YAP is essential for identity of BEC and acts as a mechanical sensor of bile canaliculi remodeling during liver regeneration [65, 146]. In response to liver injury, YAP/TAZ get activated mainly in the portal region and induce activation of quiescent biliary epithelial cells or metaplasia of hepatocytes into proliferating progenitors, required for DR-dependent liver regeneration [62, 65, 147]. Moreover, periportal hepatocytes which are close to the portal space, are specialized in the biosynthesis of lipids, known activators of YAP/TAZ, thus metabolically fueling their activation [148]. The CD44/RhoA as well as the integrin/FAK/Src axis act as upstream inputs to control YAP/TAZ activation in HPC during liver regeneration [145, 149]. Importantly, increased mechano-transduction in vivo by inhibition of Capzb (capping actin protein of muscle z-line subunit beta) leads YAP activation and HPC expansion around the portal area [150]. Moreover, increased stiffness induced by collagen accumulation during MASH progression, mechanically activates TAZ in vivo leading to MASH progression to HCC [151].

In summary, the specialized mechanics of the periportal zone influence DR activation and propagation, while changes in portal tissue mechanics primarily activate HPCs through regulation of the mechano-sensitive YAP/TAZ pathway.

### Role of the portal cell ecosystem in ductular reaction activation

**Fig. 4:**
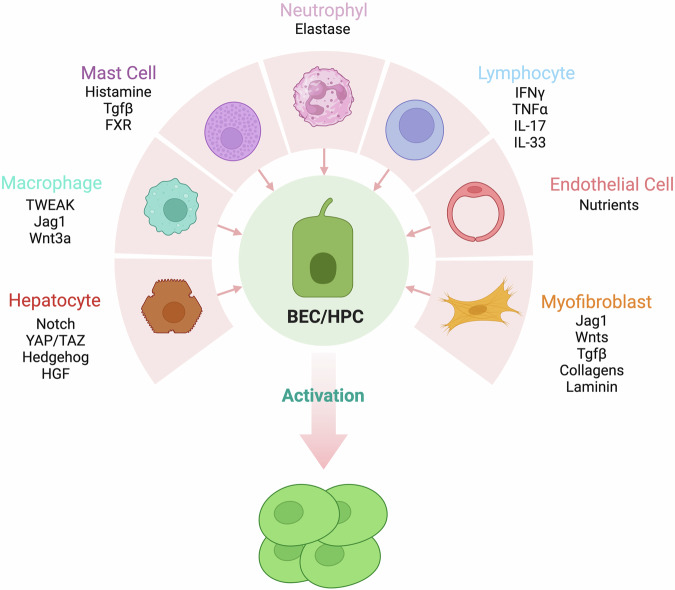
The cellular ecosystem governing DR. Upon liver injury, various cell types orchestrate the activation of biliary cells or HPC, contributing to the establishment of a ductular reaction. Neutrophils, lymphocytes, mast cells, macrophages, endothelial cells, hepatocytes, and myofibroblasts release specific signals—including cytokines, growth factors, and extracellular matrix components—that modulate HPC behavior. These interactions collectively drive HPC activation, proliferation, differentiation, and niche remodeling, ultimately shaping the DR response.

The portal space can be considered a complex cell ecosystem, where various cell types communicate, interact, and influence each other’s functions. The DR involves not only activation of HPC but also dynamic interactions with other cell types, including hepatocytes, hepatic stellate cells/myofibroblasts, portal fibroblasts, immune cells, and endothelial cells. In human, a 50% loss of hepatocytes is required for robust DR activation [152] and injured hepatocytes play a key role in supporting the DR, releasing paracrine factors that influence HPC activation and differentiation, such as Hh ligands and HGF [153–155], thus driving cholangiocyte activation and hepatic regeneration (Fig. 4) [72]. HSC, located in the space of Disse and responsible for ECM biosynthesis, maintain a quiescent phenotype in normal liver but they can get activated in response to multiple hepatic injuries. HSC are actively involved in DR regulation mainly controlling ECM deposition, thus creating a mechano-chemical niche for HPC activation and modulating their response to injury. HPC, in turn, can influence HSC activation through paracrine signaling, establishing bidirectional crosstalk between these cell populations. Of note, the number of HSC is associated with the DR stage [28]. In addition, HSC promote differentiation of HPC through inducing the Notch and Hh pathways, thus leading to the regeneration of bile ducts. In a recent study, Cordero-Espinoza et al. revealed that a specific group of SCA1^+^ periportal mesenchymal cells plays a dual influence on DR. The authors found that BEC proliferation can be either induced by soluble factors or completely abolished by contacts with mesenchymal cells, partially mediated by the Notch signaling, thus highlighting the importance of both soluble factors and physical cell-cell contacts as key regulatory cues that govern liver regeneration [156].

Immune cells, such as macrophages and lymphocytes, are integral components of the liver microenvironment and inflammation during liver injury can significantly impact the DR. Immune cells release cytokines and growth factors that can promote or inhibit HPC activation and differentiation, shaping the outcome of the DR and liver regeneration. Infiltrated portal macrophages induce HPC proliferation in mouse liver via secretion of the TNF family member called TWEAK (TNF-like weak inducer of apoptosis), acting as a selective HPC mitogen through its receptor Fn14 expressed in HPC. Moreover, Fn14 knockout mice exposed to CDE diet showed reduced DR in a NFkB-dependent manner [157–160]. Macrophages were found to promote HPC differentiation into hepatocytes in the DDC diet mouse model, and the Wnt/β-catenin pathway was the key mechanism in this process. After phagocytosis of the hepatocyte debris, macrophages increase the expression and secretion of Wnt3a, activating the β-catenin pathway in HPC and inducing differentiation into hepatocytes [47, 161]. In MASLD patients, macrophages increase significantly in the DR area, and macrophage infiltration correlates with fibrosis stage, indicating the potential role of the DR-associated macrophages in disease progression [162]. Kupffer cells, the hepatic resident macrophages, have been found to modulate the invasive behavior of DR cells, through modulation of the density of extracellular matrix as well as via Notch and Wnt signaling pathways [47, 163].

Recent studies suggest that the development of chronic liver diseases is influenced by the interaction of DR cells and mast cells (MC). MC can promote MASLD progression by activating Kupffer cells and HSC through histamine release or by direct secretion of TGF-β1. Inhibiting MC has been shown to effectively reduce DR and hepatobiliary damage [164–167]. FXR signaling in MC has been linked to liver injury and DR in cholestasis models [168]. However, the exact mechanism of how MC influence HPC activation remains unclear.

In alcoholic hepatitis, cells involved in DR exhibit an inflammatory profile actively attracting neutrophils mainly via the inflammatory mediator CXCL5 and very recent research unveiled a functional role of DR-associated neutrophils (DRANs) in chronic liver diseases, contributing to maladaptive tissue healing through elastase secretion [25, 94].

The role of lymphocytes in DR is poorly understood. Genetic experiments using immunodeficient mice and adoptive transfer of T cells point to a major role for natural killer (NK) and T cells in HPC expansion. In wild-type mice, HPC proliferation is accompanied by an intrahepatic inflammatory response, with lymphocytes producing T_H_1 proinflammatory cytokines that stimulate a DR through local secretion of IFN-γ and TNF-α [169]. During biliary injury, Hh pathway activation has been found to induce cholangiocyte production of chemokines that recruit NK cells to portal tracts [153, 170]. Finally, biliary proliferation is controlled by lymphocytes through IL-33 and IL-17 [171, 172].

DR often involves increased surrounding vascular structures, with DR cells expressing angiogenesis-related genes via Slit2-Robo1 and Endothelin pathways, in liver injury mouse models [173–175]. In this context, bile duct ligation leads to an expansion of the peribiliary plexus to support the increased biliary volume and nutrient supply. Notably, PBC patients show correlation between DR-induced angiogenesis and VEGF-expressing hepatic progenitor cells [176]. DR cells secrete angiogenic factors (VEGF-A, PDGF-B, TGF-b2, endothelin-1), often in synergy with portal myofibroblast-produced VEGF-A, driving biliary repair remodeling. A recent study has identified a therapeutic role for VEGFA in promoting progenitor-to-hepatocyte conversion in acute and chronic liver diseases [177]. These findings imply HPC-endothelial cell communication mediated by autocrine and paracrine VEGF effects [175].

Thus, understanding the intricate interaction between HPC and the liver cell ecosystem is crucial to deciphering DR mechanisms and their impact on regeneration and disease (Fig. 4).

### In vivo and in vitro models to study DR

In vivo and in vitro models of DR provide valuable tools for studying the behavior of HPC and investigating the cellular and molecular processes involved in this pathological response and allow to assess the functional consequences of DR on liver fibrosis, carcinogenesis, and overall liver function.

The CDE model utilizes a choline-deficient diet and ethionine to induce liver damage and promote DR and HPC expansion. After three weeks of CDE feeding, the liver shows signs of steatosis, hepatocellular injury, and DR cells expansion in the portal area and gradually invading the parenchyma. These DR cells exhibit the capacity to differentiate into hepatocytes, although only a small proportion of hepatocytes are derived from DR in this model. In the CDE model, DR cells have only a vague or no lumen, and comprise small elongated cells with little cytoplasm extending in the periportal parenchyma and associated with dense collagen fibers recapitulating the HPC activation typical of MASLD and hepatitis [178]. The DDC model involves a diet enriched with 3,5-diethoxycarbonyl-1,4-dihydrocollidine, causing accumulation of protoporphyrin and biliary damage. After 3 weeks of DDC feeding, dysmorphic cholangiocytes proliferate in the portal area, leading to the expansion of DR within the portal mesenchyme. The DR pattern observed in DDC livers resembles that of PSC and PBC, where DR proliferation is abundant within the portal area and is accompanied by periportal fibrosis.

Another commonly used in vivo model of the DR is the bile duct ligation (BDL) model, where obstruction of the bile duct leads to cholestasis, inducing strong proliferation of cholangiocytes and HPC, resulting in extensive DR, portal inflammation and rapid establishment of biliary fibrosis [179]. This model allows for the study of HPC activation and differentiation in the context of portal injury and fibrosis. The genetically-modified Mdr2 knockout mouse model is widely utilized to study DR. In this model, the Mdr2 gene, which encodes for a phospholipid transporter essential for biliary phospholipid secretion, is knocked out. This disruption leads to impaired biliary excretion and results in the accumulation of toxic bile acids within the liver, triggering liver injury and the activation of DR. The Mdr2 knockout model provides valuable insights into the pathogenesis of liver diseases involving DR and biliary injury

The induction of acute liver damage using acetaminophen (APAP), also known as paracetamol, represents a frequently employed experimental approach for inducing acute liver injury in mice. Given that APAP prompts a DR, the similarity in mechanisms and histology of acetaminophen-induced injury between mouse and human livers renders it a valuable model for studying liver regeneration and the involvement of progenitor cells in this phenomenon [180].

In vitro models, on the other hand, involve the culture of isolated liver cells or cell lines under defined conditions. These models allow for a more controlled examination of the cellular and molecular mechanisms underlying the DR. Primary BEC or HPC can be isolated and cultured, providing a platform to investigate their behavior, differentiation potential, and responses to various stimuli [181–183]. Co-culture systems, where HPC are cultured with other cell types such as hepatocytes or hepatic stellate cells, can mimic the in vivo cellular environment and facilitate the study of paracrine signaling and bidirectional crosstalk. More recently, advancements in tissue engineering have led to the development of organ-on-a-chip, three-dimensional (3D) assembloids and organoid models, that better mimic the native liver tissue architecture and microenvironment. These models allow for the study of HPC behavior and differentiation in a more physiologically-relevant context.

Cholangiocyte organoids, derived from human and mouse adult liver tissue or induced pluripotent stem cells (iPSCs), offer a valuable in vitro model to study DR [53, 184–186]. These organoids, representing stable HPC cultures, express stem-cell genes and recapitulate the profiles of regenerative HPC in injured mouse liver. They can differentiate into functional hepatocytes in vitro and show engraftment potential in the damaged mouse and human liver upon transplantation, demonstrating their high plasticity [16, 184]. Cholangiocyte organoids from cirrhotic patients mimic liver DR and faithfully recapitulate DR-associated inflammatory phenotype [94]. Cholangiocyte organoids can repair different regions of the biliary tree when transplanted, making them a promising tool for regenerative medicine and studying DR in vitro [16]. The recent implementation of hydrogels to replicate the mechanical characteristics of the fibrotic liver microenvironment in 3D cultures, represents a significant advancement in this field [145]. This advanced model enables a more comprehensive investigation of DR in a highly relevant and realistic context, providing valuable insights into liver disease progression and potential therapeutic strategies.

### Therapeutic implications

The DR is emerging as a promising therapeutic target for managing liver diseases. Manipulating this response can yield substantial benefits, including promoting liver regeneration, mitigating or preventing fibrosis, and potentially preventing liver cancer. Approaches to modulate the DR involve influencing specific signaling pathways, growth factors, and cytokines that govern HPC activation and differentiation. By targeting the underlying cellular and molecular processes, therapeutic strategies could be designed to control the activation, expansion, and differentiation of the DR. The recent studies suggesting that mature hepatocytes are the principal source of newly formed liver cells, with the DR cells contributing minimally to true parenchymal regeneration [187], together with the evidence that expanding DR cells sustain an inflammatory and profibrogenic milieu, has directed the focus of translational investigations toward mitigating or preventing the DR itself.

Several pharmacological interventions demonstrate that dampening the DR can yield tangible therapeutic benefits. In the Mdr2^–/–^ mouse, treatment with 24-norursodeoxycholic acid (norUDCA) substantially reduces ductular proliferation and inflammation, leading to less portal fibrosis and improved liver pathology [188]. In this context, genetic and pharmacological inhibition of Farnesoid X receptor (FXR), have also shown efficacy in downregulating ductular proliferation and reducing hepatic fibrogenesis, reflecting broad antifibrotic and anti-DR effects in cholestatic contexts [189, 190]. Likewise, in the DDC-fed mouse, pharmacological Hedgehog pathway inhibition diminishes DR and collagen deposition, thereby curbing fibrosis [191]. Additionally, administration of S63845, a proapoptotic BH3-mimetic therapy, significantly reduced the DR-cell population and markers of inflammation and liver fibrosis in Mdr2^–/–^ mice, while targeted removal of senescent cholangiocytes via AP20187 or fisetin leads to a reduction in the expression of inflammatory, fibrotic, and senescence markers associated with the disease [17, 192].

In conclusion, targeting the DR in liver diseases carries significant therapeutic potential. Modulating it, through pharmacological approaches, offers promise for regeneration, fibrosis alleviation, and cancer prevention. However, there are currently no FDA-approved therapies designed to target the DR in a highly specific manner. This highlights the need for deeper mechanistic insights into how reactive cholangiocytes expand, with the aim to identifying new vulnerabilities within the DR. Such strategies may not only improve liver architecture and function but also help preventing tumorigenesis in the chronically injured liver, such as in PSC patients.

### Future perspectives and open questions

Despite significant advances in the field, there remain numerous open questions regarding the DR that call for deeper investigation. First, while it is known that various etiologies can trigger a DR, it is not fully understood whether these divergent insults give rise to fundamentally distinct DR cell populations or whether they represent different states of a common progenitor pool. Clarifying the lineage relationships and plasticity of these cells is essential, as such insights could reveal universal versus disease-specific therapeutic targets. Second, the dual nature of the DR remains a major conundrum. Determining the molecular and cellular cues that tip the DR from a beneficial, reparative role to a maladaptive, proinflammatory state is thus a central priority. In particular, dissecting the interactions between DR cells and other key players—such as hepatic stellate cells, macrophages, and diverse immune cell populations—will help illuminate the mechanisms by which the microenvironment shapes DR outcomes. Similarly, the role of the DR in carcinogenesis, particularly in creating a permissive environment for the development of HCC and CCA, raises important questions about the genetic and epigenetic changes that underlie this transition. Third, a suite of powerful new technologies now promises to bring unprecedented detail to DR research. Single-nucleus RNA sequencing (snRNA-seq) can identify rare or transitional cell states within a complex tissue, multiplex imaging can reveal the spatial organization and cell-cell interactions, and spatial transcriptomics can map gene expression to specific anatomical niches. Together, these tools may help researchers clarify spatially resolved mechanisms of distinct DR phenotypes and regulatory networks. Fourth, an important step forward to address these questions, will be the development of advanced multi-cellular in vitro models—such as organoids grown in defined hydrogels or precision-cut liver slices, that include a native ECM components and intact histological architecture. These organotypic cultures may capture critical paracrine and biomechanical signals that are lost in simpler monolayer systems, enabling more accurate dissection of DR mechanisms, as well as preclinical screening of therapeutic strategies aimed at controlling its proinflammatory and profibrogenic effects. In terms of clinical application, the potential to identify reliable biomarkers derived from DR cells, such as secreted cholangiokines, could revolutionize the non-invasive diagnosis and monitoring of liver disease progression. Finally, the biomechanical properties of the extracellular matrix and their influence on DR activation and propagation are emerging and present intriguing possibilities for therapeutic intervention, particularly in modulating mechano-transduction to support regeneration while limiting pathological effects.

A comprehensive understanding of these unresolved questions will not only enhance our knowledge of DR biology but also pave the way for precision medicine approaches that can selectively modulate DR depending on the disease context—either promoting its regenerative role in liver repair or, more realistically, inhibiting its pro-fibrotic and tumorigenic effects in chronic liver disease.
